# Enhanced Delivery of 5-Aminolevulinic Acid by Lecithin Invasomes in 3D Melanoma Cancer Model

**DOI:** 10.1021/acs.molpharmaceut.3c00494

**Published:** 2023-09-27

**Authors:** Antonio Gaballo, Andrea Ragusa, Concetta Nobile, Nunzia Gallo, Luca Salvatore, Clara Piccirillo, Alessia Nito, Annalisa Caputo, Gabriella Guida, Alfredo Zito, Raffaele Filotico, Alessandra Quarta

**Affiliations:** † Consiglio Nazionale delle Ricerche, Institute of Nanotechnology, via Monteroni, Lecce, 73100, Italy; ‡ Department of Biological and Environmental Sciences and Technologies, University of Salento, via Monteroni, Lecce, 73100, Italy; § Department of Engineering for Innovation, University of Salento, via Monteroni, Lecce, 73100, Italy; ∥ Typeone Biomaterials Srl, Muro Leccese, Lecce, 73036, Italy; ⊥ Department of Basic Medical Sciences Neurosciences and Sense Organs, University of Bari, Bari, 70124, Italy; # Pathology Department, IRCCS Istituto Tumori “Giovanni Paolo II”, Bari, 70124, Italy; ○ Dermato-Oncology Unit, IRCCS Istituto Tumori “Giovanni Paolo II”, Bari, 70124, Italy; □ Section of Dermatology and Venereology, Department of Precision and Regenerative Medicine and Ionian Area (DiMePRe-J), University of Bari “Aldo Moro”, Bari, 70124, Italy

**Keywords:** photodynamic therapy, 3D melanoma spheroids, invasome, 5-aminolevulinic acid, transdermal delivery, ROS

## Abstract

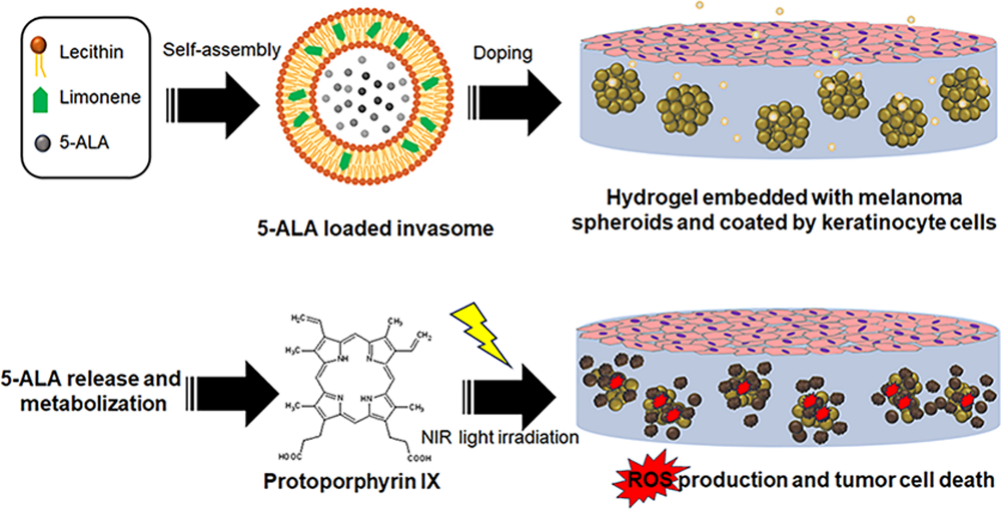

Photodynamic therapy (PDT) is a noninvasive therapeutic approach for the treatment of skin cancer and diseases. 5-Aminolevulinic acid is a prodrug clinically approved for PDT. Once internalized by cancer cells, it is rapidly metabolized to the photosensitizer protoporphyrin IX, which under the proper light irradiation, stimulates the deleterious reactive oxygen species (ROS) production and leads to cell death. The high hydrophilicity of 5-aminolevulinic acid limits its capability to cross the epidermis. Lipophilic derivatives of 5-aminolevulinic acid only partly improved skin penetration, thus making its incorporation into nanocarriers necessary. Here we have developed and characterized 5-aminolevulinic acid loaded invasomes made of egg lecithin, either 1,2-dilauroyl-*sn*-glycero-3-phosphocholine or 1,2-dioleoyl-*sn*-glycero-3-phosphocholine, and the terpene limonene. The obtained invasomes are highly thermostable and display a spherical morphology with an average size of 150 nm and an encapsulation efficiency of 80%; moreover, the *ex vivo* epidermis diffusion tests established that nanovesicles containing the terpene led to a much higher skin penetration (up to 80% in 3 h) compared to those without limonene and to the free fluorescent tracer (less than 50%). Finally, in vitro studies with 2D and 3D human cell models of melanoma proved the biocompatibility of invasomes, the enhanced intracellular transport of 5-aminolevulinic acid, its ability to generate ROS upon irradiation, and consequently, its antiproliferative effect. A simplified scaffold-based 3D skin model containing melanoma spheroids was also prepared. Considering the results obtained, we conclude that the lecithin invasomes loaded with 5-aminolevulinic acid have a good therapeutic potential and may represent an efficient tool that can be considered a valid alternative in the topical treatment of melanoma and other skin diseases.

## 1 Introduction

Among skin cancers, melanoma is one of the most aggressive due to its ability to rapidly metastasize. Cutaneous melanoma originates in the deeper part of the epidermis, which is made of three layers: the outer stratum corneum (SC; keratinocytes), basal cells, and melanocytes.^1^ The uncontrolled proliferation of melanocytes, due to mutations or uncontrolled activation of cell signaling pathways, leads to the onset of melanoma.^2^

The primary clinical treatment of melanoma includes surgical resection, which is effective only in the very early stages.^3^ Other therapeutic approaches include chemotherapy, targeted therapy, radiotherapy, and immunotherapy, which proved to be the most effective when tumor metastasis occurs. However, all these therapeutic strategies have three general main limitations, which are adverse effects, therapeutic resistance, and low economic sustainability (high costs). Therefore, there is still the need to develop new more effective and more affordable therapeutic systems for primary melanoma lesions.^4−7^

A more innovative treatment of nonmetastatic melanoma is photodynamic therapy (PDT).^8,9^ PDT is a minimally invasive medical treatment with the advantages of high selectivity and direct access to the target area (melanoma cells) with almost no side effects.^10^ This approach is based on the use of a photosensitizer that is administered topically and selectively concentrated around the malignant tissue; when irradiated by a light with an appropriate and specific wavelength, the photosensitizer is activated and can produce cytotoxic reactive oxygen species (ROS). These molecules can damage biomolecules, leading to cell apoptosis or necrosis thus causing the destruction of cancer cells followed by inflammation; this, in turn, could possibly further stimulate the host immune system.^11,12^

Among various photosensitizers, most clinical studies focused on protoporphyrin IX (PpIX) precursors, particularly 5-aminolevulinic acid (5-ALA).^13^ 5-ALA is a stable small nonproteinogenic amino acid, precursor of Heme biosynthesis. In cells, during the Heme synthesis pathway, this molecule is converted into PpIX, a photosensitizer excited by light between λ = 405 (violet) and λ = 633 nm (red).^14^ Additional interesting properties are attributed to 5-ALA, such as its fast elimination inside the body; moreover, as it accumulates in not many nonmalignant tissues, it can provide a good signal without much noise for tumor imaging. Also, being an endogenous human metabolite, its use is not accompanied by a toxic side effect.^15,16^ Although ALA can be systemically administered, the topical administration is preferred since the intravenous route has the disadvantage of causing a long-lasting photosensitivity, which means patients have to avoid exposure to light for about 48 h after treatment.

The main limiting characteristic of 5-ALA is its poor permeability to the skin. As this molecule is a hydrophilic zwitterion and the stratum corneum is very hydrophobic, its passage through it is difficult.

Nanotechnology has recently developed several nanocarriers for transdermal drug delivery. Their structure and transdermal delivery mechanism vary with the composition and the structural features.^17^ As an example, recently, nanocomplexes based on poly(amidoamine) dendrimers loaded with 5-ALA have been tested in the PDT of melanoma cancer.^15^ Among the various typologies of nanosystems, those based on lipid formulations (lipid nanoparticles and lipid nanovesicles) have been widely studied.^18,19^

Lipid nanoparticles are solid particles, mainly encapsulating drug molecules in a nonaqueous core; some of them have been tested for the delivery of lipophilic photosensitizers, such as Verteporfin and chlorin e6.^20^

Differently from lipid nanoparticles, lipid-based nanovesicles are spherical vesicles with one or more lipid bilayer and an aqueous inner core, in which hydrophilic drugs can be encapsulated. Liposomes, the most common lipid-based nanovesicle, are generally formed with phospholipid molecules and cholesterol.^21^ However, they are unable to permeate the inner layers of skin, and the accumulation has been mainly observed in the upper part of the epidermis because of its relatively low fluidity.^18,19^ Therefore, great efforts have been made to improve skin penetration by developing other lipid-based nanovesicles with different compositions.^22,23^ Among them, invasomes contain ethanol and terpene in their phospholipid bilayer; the addition of terpenes creates deformable vesicles, and this elasticity confers more efficient transdermal penetration properties to the soft vesicles.^24,25^

Although several applications of invasomes have been described,^21^ to our knowledge, no data are available on the 5-aminolevulinic acid-loaded invasomes and on their employment in the topical treatment of melanoma. Since invasomes must seep through the skin, vesicle size is a fundamental element to take into account in their preparation.

For this reason, the current study aims to develop biocompatible, small-sized invasomes with a high skin penetration efficiency made of egg lecithin, either 2-dilauroyl-*sn*-glycero-3-phosphocholine or 1,2-dioleoyl-*sn*-glycero-3-phosphocholine, and limonene to encapsulate 5-ALA and deliver it to melanoma cells. Several conditions were tested to optimize the nanovesicle size and encapsulation efficiency. The in vitro degradation profile of the invasomes was characterized by using cells lysates. Cellular studies with 2D and 3D cellular models of melanoma were performed to assay the biological response to the administration of 5-ALA-loaded invasomes. Thus, a simplified skin model made of human keratinocytes layered onto an agarose matrix embedding melanoma spheroids was developed. Finally, the diffusion kinetic of the nanovesicles through ear pig skin was evaluated showing that the invasomes penetrate to a higher extent than nanovesicles without the terpene.

## 2 Materials and Methods

For the synthesis of the nanovesicles, the chemicals used were 1,2-dilauroyl-*sn*-glycero-3-phosphocholine (DLPC), 1,2-dioleoyl-*sn*-glycero-3-phosphocholine (cisPC), and egg yolk lecithin, all purchased from Avanti Polar Lipids. Ethanol was acquired at VWR Chemicals. The ultrapure water was obtained from the Ultrapure water purification system. Agarose, limonene, fluorescamine, rhodamine 101, 5-aminolevulinic acid (5-ALA), thiazolyl blue tetrazolium bromide, and 2′,7′-dichlorofluorescein diacetate were purchased from Sigma-Aldrich. LIVE/DEAD Viability/Cytotoxicity Kit (L&D) assay was purchased from Thermo Fisher Scientific. The human keratinocyte cells, namely, HaCat was obtained from American Type Culture Collection (ATCC). The human melanoma cell line (HBL) was a gift from Prof. G. Ghanem, Université de Bruxelles, Belgium.

### 2.1 Synthesis of the Lipid Nanovesicles

The nanovesicles were prepared by the solvent addition method.^26^ The lipids (9 mg) and limonene (1 mg) were dispersed in 1.5 mL of ethanol and transferred to a glass vial. Using a syringe pump, 5 mL of ultrapure water was added (2.5 mL/min rate) to each vial while vigorously stirring the sample using an orbital shaker. Finally, the samples were left stirring at 400 rpm in an orbital shaker prior to being ultrasonicated for 20 min (25% power intensity) in an ice bath (SONOPLUS HD3100 operating at 20 kHz). Then, the samples were dialyzed (Sigma-Aldrich Dialysis Tubing Cellulose Membrane of 25 mm width and 14000 kDa molecular cutoff) overnight under stirring at 4 °C.

To encapsulate the fluorescent dye, rhodamine 101, 30 μL of rhodamine (2 mM in ethanol) was added to the lipid mixture. Then the preparation protocol was followed, as described.

#### 2.1.1 Encapsulation of 5-ALA

To encapsulate the prodrug 5-ALA into the nanovesicles, the protocol was slightly modified in order to increase the encapsulation efficiency.

5-ALA, as a zwitterion, possesses two ionization constants (p*K*_a_ = 4.0 and p*K*_b_ = 8.2). According to the literature, nonionized 5-ALA can diffuse through the lipid bilayers freely, while protonated 5-ALA cannot diffuse through the lipid bilayer freely.^27^ Therefore, a mechanism of active loading through a pH gradient can be used to entrap more molecules of 5-ALA inside the vesicles. For this aim, the nanovesicles were prepared using an acidic solution (phosphate buffer solution, pH 4.5) instead of ultrapure water. At pH 4.1, the drug is in its protonated form. When the pH of the medium is brought to neutral by adding NaOH 1 M, 5-ALA turns from the protonated status to the nonionized status making them able to cross the bilayer of the nanoparticles. When a molecule of 5-ALA enters a vesicle, it turns into its pronated form again due to the acidic environment. Consequently, the molecule loses its ability to cross the bilayer and stays entrapped inside the nanoparticle.^27^

In detail, the mixture of phospholipids dissolved in ethanol was prepared and split into three vials. In each vial, 5-ALA (5 mg/mL dissolved in ultrapure water) was added. The samples were then placed in the orbital shaker, followed by the dropwise addition of 5 mL of phosphate buffer solution (pH 4.5). The sample was left under stirring at 700 rpm in an orbital shaker for 30 min, then NaOH (1 M) was added until the pH of the sample reached a value close to 7.4. Finally, the sample was stirred overnight and then dialyzed. The final suspension of the nanovesicles was stored in the fridge at 4 °C. The stability of the nanovesicles was monitored over time for up to 30 days.

### 2.2 Characterization of the Nanovesicles

#### 2.2.1 Encapsulation Efficiency of Rhodamine 101

To calculate the encapsulation efficiency (EE) of rhodamine, an indirect measurement method was used. The water resulting from the dialysis was recovered, and its fluorescence was measured by a fluorimeter (Cary Eclipse). Plotting the fluorescence value on a Rhodamine calibration curve, the concentration of the not encapsulated fluorophore was determined. Then, the encapsulated amount was estimated and reported as EE according to the following formula:
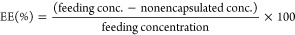


#### 2.2.2 Estimation of 5-ALA Encapsulation

**Scheme 1:**
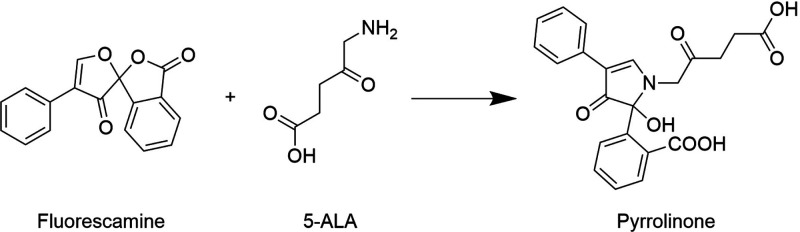
Reaction between Fluorescamine and 5-ALA

To detect 5-ALA rapidly and accurately, a modified fluorescamine assay was established. The method is based on the conversion of 5-ALA into a fluorescent derivative after reacting with fluorescamine, as represented in Scheme 1. As in the case of rhodamine, the nonencapsulated molecule was first quantified, and then, by subtracting the feeding amount, the encapsulated concentration was obtained.

The protocol consists of mixing 300 μL of the sample, 270 μL of fluorescamine (0,1% in acetonitrile), and 45 μL of borate buffer solution (pH 8.0). The mixture was vortexed for 5 s and put into a dark environment for 5 min for the reaction to occur. The fluorescamine assay was performed with dialysis water, with the feeding solution of 5-ALA, and with all the standard solutions prepared for the calibration curve. Indeed, as already described in the case of rhodamine, a calibration curve with 5-ALA (concentrations ranging from 0.5, 1, 5 to 25 μg/mL) was prepared. After the incubation time, 200 μL of each mixture was transferred to a multiwell plate. The fluorescence of the mixture was then measured (excitation wavelength at 380 nm and emission wavelength at 480 nm) using a multiwell reader CLARIO starPlus BMG LABTECH.

Finally, to achieve the value of the EE the following formula was applied.
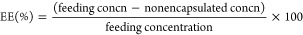


The loading capacity (LC) corresponds to the mass of the drug entrapped per unit of mass of the nanovesicles.

First, the nanovesicles loaded with the drug were lyophilized, and their dry mass was determined. The mass of the sample entrapped was calculated by multiplying the feeding drug mass by the EE of the sample. Successively, to determine the LC, the following formula was applied.
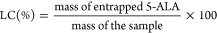


The mass of the nanovesicles was determined by measuring the dry weight of the nanovesicles after the lyophilization.

#### 2.2.3 Release of 5-ALA and Rhodamine 101 from the Nanovesicles

The release assay was initially performed with the nanovesicles loaded with 5-ALA. The samples were incubated in PBS solution at two different pH values, 7.4 and 4.5, respectively, at 37 °C. Four incubation points were considered, 30 min, 1, 2, and 4 h. At the end of the incubation point the samples were centrifuged through Amicon filters (10000 kDa MW Cutoff) and the solutions collected in the lower compartment were used to estimate the released prophotosensitizer by fluorescamine assay.

In the case of the invasomes loaded with rhodamine 101, the release assay was performed upon incubation of the samples with the freshly prepared cell lysate of melanoma cells freshly prepared. In detail, 2 mL of fluorescent invasomes (diluted in PBS pH 7.4) were added to 200 μL of cell lysate and left under incubation at 37 °C for 10 min. Soon after, the suspension was filtered through 0.2 μm filters, and the collected solution measured by the fluorimeter.

### 2.3 Diffusion of the Nanovesicles into Ex Vivo Pig Ear Skin

**Scheme 2:**
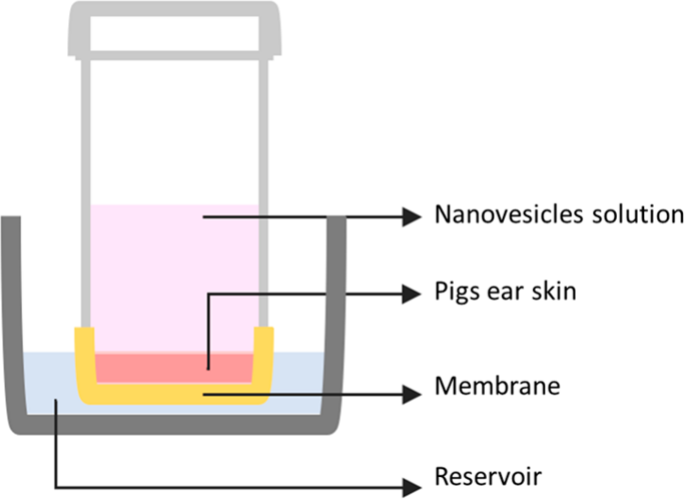
Representation of the Setup Used To Study the Diffusion of the Nanovesicles into Skin

The initial tests of the particles’ diffusion into the skin were done in samples of ex vivo pig ear skin. Scheme 2 represents the apparatus developed to perform these tests, inspired by a Franz diffusion cell.

The apparatus consists of a tube equipped with a membrane at the bottom over which the pig ear skin is glued to the edges. Disks of pig skin with the same diameter as the internal wall of the tube were previously cut. In the upper part of the tube, the fluorescent samples (either nanovesicles/Rho101 or free rhodamine) were loaded, and the fluorescence decay was measured over time (time 0, 15, 30 min, 1, 2, and 3 h).

The percentage of fluorescence decrease was calculated according to the following formula:
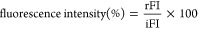
where iFI is the initial fluorescence intensity and rFI is the residual fluorescence intensity. After the fluorescence analysis, the skin disks were recovered and observed under a fluorescence microscope.

Additionally, the skin samples were cut into small pieces and placed into a dialysis bag. The samples were dialyzed in water for 24 h prior to recording the fluorescence signal of either the nanovesicles and the free Rhodamine released from the pig skin.

### 2.4 Cell Culture

HBL melanoma cells were a gift from Prof. G. Ghanem, Université de Bruxelles, Belgium.^28^ Human keratinocytes Hacat were supplied by ATCC. Both types of cells were grown in DMEM supplemented with 10% fetal bovine serum (FBS), 2 mM glutamine, 100 IU/mL penicillin, and 100 μg/mL streptomycin in an incubator at 37 °C in a humidified atmosphere with 5% CO_2_.

#### 2.4.1 Preparation of Agarose Hydrogels for the Growth of Melanoma Spheroids and the Keratinocyte Layer

Agarose was dissolved in sterile phosphate-buffered saline (PBS, pH 7.4) and heated on a hot plate to obtain a 1% (w/v) stock solution. The warm agarose solution (at around 45 °C, the gel point is at 36 °C) was added to the cell suspension to obtain 0.18% agarose and 50000 cells/mL. The agarose/cell suspension was gently stirred with a glass rod, rapidly transferred in each well of a 24 wells plate, and allowed to gel at room temperature. After gelation, 1 mL of DMEM was added to the cell-embedded hydrogels, and the plate was transferred into the incubator. The medium was changed every 4 days, and the growth of the spheroids was monitored over time. After 7 days of growth, Hacat cells (25000 cells) were seeded over the agarose hydrogel and allowed to adhere to form a confluence layer.

### 2.5 Cellular Assays

#### 2.5.1 Administration of the 5-ALA-Loaded Nanovesicles and Photodynamic Therapy

To assay the biological efficacy of the nanovesicles, 2.5 × 10^4^ cells suspended in 200 μL of culture medium were seeded into each well of 96 multiwell plates for 24 h. Then, the 5-ALA-loaded nanovesicles and free 5-ALA were added to the cell medium to reach a final concentration of 25 and 50 μg/mL, in accordance with previously published works.^5^ Empty vesicles were also added as a control sample. The cells were incubated for 3 h. Then the medium was removed and replaced with 100 μL of Leibovitz medium, and the cells were irradiated for 15 min with a red lamp emitting at 630 nm (power supply 11 V).

#### 2.5.2 MTT Assay

The cytocompatibility of the nanovesicles and the cytotoxic effect of the combined 5-ALA/PDT treatment were determined using the thiazolyl blue tetrazolium bromide (MTT) assay. Cells were seeded in 96-well plates at a density of 2.5 × 10^4^ per well and incubated at 37 °C in 5% CO_2_. After overnight incubation, the nanovesicles were added at different concentrations (from 50 to 500 μg/mL, each point in triplicate) and incubated at 37 °C. After 3 h, the medium was removed and replaced with a serum-free medium containing 2 mg/mL MTT and incubated for 2 h at 37 °C. The MTT reagent was then removed and the formazan crystals were solubilized using dimethyl sulfoxide. The absorbance was read by using the CLARIO star Plus microplate reader (570 nm). The absorbance of the vehicle control was subtracted, and the percentage control was calculated as the absorbance of the treated cells/control cells.

#### 2.5.3 Measurement of Intracellular Reactive Oxygen Species (ROS)

To detect the changes in intracellular ROS levels, 2′,7′-dichlorofluorescein diacetate (DCFH-DA, Sigma-Aldrich) staining was used.^29^ DCFH-DA is a stable, fluorogenic, and nonpolar compound that can readily diffuse into the cells and get deacetylated by intracellular esterases to a nonfluorescent 2′,7′-dichlorodihydrofluorescein (DCFH) that intracellular ROS later oxidizes into highly fluorescent 2′,7′-dichlorofluorescein (DCF). The intensity of the fluorescence is proportional to the intracellular ROS levels. After the treatment with encapsulated and free 5-ALA and the light irradiation (635 nm wavelength, 25 mW cm^–2^), the cells were washed once with fresh DMEM and twice with 1× PBS. Then, they were incubated with DCFH-DA (10 μM) for 30 min and rinsed with PBS. Representative fluorescent images for each well using the green fluorescent protein (GFP) channel were taken on an Evos M7000 fluorescence microscope. After images were taken, PBS was removed, and a radioimmunoprecipitation assay (RIPA) buffer was added to each well. The collected cells were incubated at −80 °C for 20 min and then centrifuged at 21130 g for 10 min at 4 °C. The collected supernatant was transferred to a black 96-well plate, and the fluorescence intensity was measured using the CLARIO star Plus microplate reader at an excitation wavelength of 485 nm and an emission wavelength of 530 nm. After fluorescence recording, 5 μL of supernatant was transferred to a transparent 96-well plate containing 195 μL of the protein assay solution to measure the protein concentration using the BCA assay. The fluorescence intensity was normalized to the protein concentration.

#### 2.5.4 Live/Dead Assay

Viability of the spheroids was investigated by using a live/dead assay kit (Thermo Fisher Scientific Inc., Waltham, MA, U.S.A.). Briefly, the activity of intracellular esterase induces nonfluorescent, cell-permeant calcein acetoxymethyl ester to become fluorescent after hydrolysis, giving a green fluorescence to the viable spheroids. On the ethidium, the homodimer enters and binds to nucleic acids only in damaged cells, producing a red fluorescence that indicates dead cells. The assay was performed after 7 days of growth of the spheroids into the hydrogel. In detail, the medium was replaced with fresh medium containing either the nanovesicles or 5-ALA (50 μg/mL), and after 3 h of incubation, the embedded spheroids were washed with PBS before light irradiation. Then, a phosphate buffer solution containing the reagents of the assay was added, and the plate was kept incubated at 37 °C for 1 h. Finally, the solution was replaced with fresh PBS before imaging the samples under a fluorescence microscope (EVOS Floid Cell Imaging Station, ThermoFisher, Waltham, MA, U.S.A.).

## 3 Results

### 3.1 Invasomes Synthesis and Characterization

**Scheme 3:**
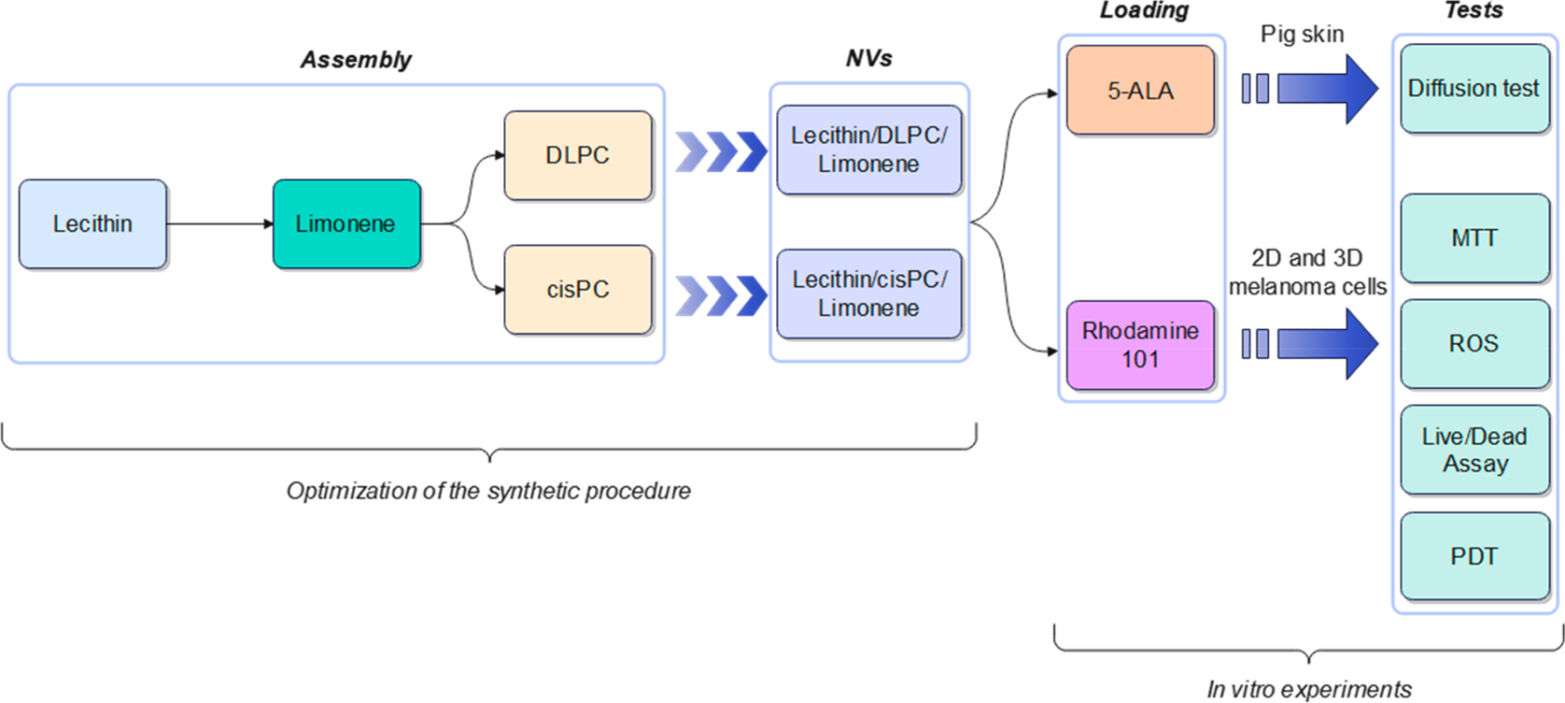
Outline of the Study Showing the Synthetic Procedure Used for the Preparation of the Invasomes and the Applicative Tests with 2D and 3D Melanoma Cells

Scheme 3 summarizes the main steps of this study, including the development and testing of 5-ALA-loaded vesicles designed for topical delivery and the PDT of melanoma.

Initial experiments were performed by preparing the invasome-like nanovesicles with just egg lecithin and a terpene. Three different terpenes were tested in parallel, limonene, eugenol, and geraniol, respectively. They were synthesized in an ethanol–water mixture by the solvent addition method, followed by ultrasonication (20 min, 25% power); the combination of the two techniques allowed to obtain high amounts of sample in a short time with controlled size distribution and with a great reproducibility.^30^ The volume of ethanol was set to 1.5 mL, as it was sufficient to dissolve the lipid mixture and better to obtain small vesicles. Table S1 reports the average size and polydispersity index (PDI) of the lecithin-terpene invasomes prepared by varying the feeding mass of terpene. By increasing the amount of terpene a slight decrease of the average diameter was detected (from 320 to 237 in the case of eugenol, from 282 to 205 in the case of geraniol and from 250 to 220 nm in the case of limonene) likely due to a compression of the lipid bilayer. The IC_50_ evaluated by a MTT assay of melanoma cells incubated for 24 h with the nanovesicles containing the different terpenes decreased from around 600 to 130 μg/mL. Based on these biocompatibility data and the average size of the resulting vesicles, 1 mg of limonene was selected for the preparation of the nanovesicles hereafter.

**Figure 1:**
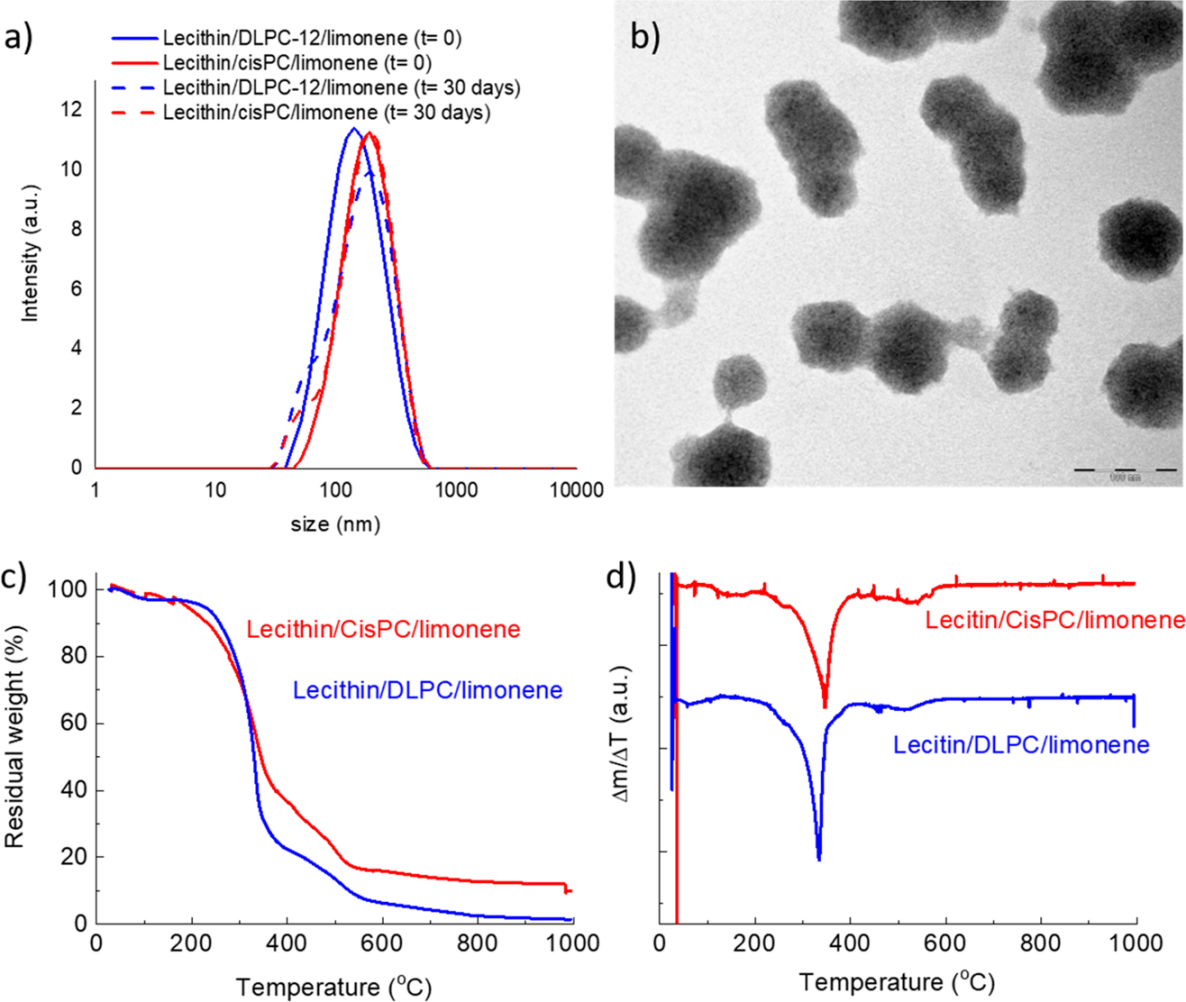
(a) DLS curve of the invasomes containing either DLPC or cisPC at *t* = 0 (solid lines) and at *t* = 30 days (dashed lines); (b) Representative TEM image of the nanovesicles composed of lecithin/DPLC/limonene. TGA curves of the two types of nanovesicles: (c) Percentage residual weight and (d) first derivative curves.

To further reduce the hydrodynamic diameter of the vesicles below 200 nm, we considered other phospholipids as additional components of the vesicles. In detail, DLPC, DMPC, LysoPC, DSPE, and cisPC were tested; they differ in the length and the unsaturation/saturation of the alkyl chain. As shown in Table S2 and Table 1, the smallest nanovesicles with the highest biocompatibility profile (IC_50_ evaluated by MTT assay of melanoma cells) were obtained in the case of DLPC and cisPC, as indeed the size of the resulting invasomes was around 120 and 140 nm, respectively. Furthermore, the values of the PDI < 0.2 indicate the homogeneous distribution of the samples. Therefore, these two phospholipids (DLPC and cisPC) were selected as additional components of the nanovesicles whose recipe consists of lecithin/limonene/DLPC or lecithin/limonene/cisPC. Table 1 also reports the surface charge of these two types of vesicles, whose value is around −15 mV, and the average size after 30 days of aging at 4 °C. The size curves of the samples are reported in Figure 1a, which also includes the curves after 30 days of aging; data show only a slight broadening of the curves upon one month storage.

**Table 1:** Size Distribution As Determined by DLS Measures and PDI of the Vesicles Made of Lecithin/DLPC/Limonene and Lecithin/cisPC/Limonene at *t* = 0 and after 30 Days^a^

sample	DLS (nm) at *t* = 0	PDI at *t* = 0	surface charge (mV)	DLS (nm) after 30 days	PDI after 30 days	IC_50_ (μg/mL)
lecithin/DLPC/limonene	125 ± 7	0.18 ± 0.1	–15.3 ± 3.5	128 ± 5	0.24 ± 0.2	495 ± 21
lecithin/cisPC/limonene	140 ± 3	0.17 ± 0.1	–16.6 ± 2.7	145 ± 1	0.22 ± 0.1	530 ± 34

a The fourth column reports the average surface charge of the two nanosystems. The last column reports the IC_50_ values as determined by the MTT assay with melanoma cells.

The morphology of the vesicles prepared with DLPC under electron microscopy (Figure 1b) shows the spherical and uniform distribution of the samples. Those containing cisPC display a similar morphology (data not shown).

The thermostability of the invasomes was analyzed by thermogravimetric measures. Figure 1c,d shows the TGA curves and their first derivatives for the nanovesicles containing either DLPC or cisPC, respectively. It can be seen that in both samples an almost complete weight loss was registered for temperature up to 600 °C; this means that the presence of either DLPC and cisPC did not affect significantly the thermal stability of the systems.

However, some differences between the two systems can be observed. Nanovesicles containing DLPC, in fact, showed a small weight loss for *T* < 100 °C and then almost no loss for temperatures up to about 250 °C; for those with cisPC, on the other hand, a more regular loss was observed for the whole temperature interval. This temperature range corresponds to the loss of water, first that adsorbed on the surface (up to 100 °C) and, successively, that incorporated into the systems (up to 200–250 °C). These data indicate that when cisPC is employed, more water is incorporated into the NPs structure, likely due to the presence of two carbon–carbon double bonds in the molecule.

The greater weight loss for both types of vesicles was registered for 250 °C < *T* < 400 °C, with a further loss for 400 °C < *T* < 600 °C. Regarding the first one, the first derivative curves show a quite sharp peak for both samples at about 330 °C; the shape of the peak indicates an abrupt weight loss. The weight loss in this temperature interval corresponds to the degradation/combustion of some organic molecules or part(s) of them; this step might be associated with the combustion of the aliphatic chain. The small difference in the temperature between the two samples could be due to the higher stability of the carbon–carbon double bond in the cisPC, which requires more energy to be broken.

The loss at higher temperature corresponds to the degradation of the rest of the molecules, i.e., phosphate, carboxylic, and aminic groups, with no major differences observed between the two systems.

Considering the DTA curves (Figure S1a), it can be seen that the major weight losses correspond to exothermic peaks; this is in agreement with TGA data as combustion is an exothermic process.

TGA tests were also performed on both systems 30 days after their preparation; the results (Figure S1b) were not significantly different from those of the freshly prepared nanovesiscles. This confirms the stability of the vesicles over time, making them a suitable tool for practical applications.

### 3.2 Encapsulation of 5-ALA

Upon optimization of the preparation protocol, 5-ALA was encapsulated into the nanovesicles by using a pH gradient, as described in the experimental section. Indeed, when 5-ALA was entrapped by diffusion into the bilayer, a modest encapsulation was detected (around 10%, data not shown).

To determine the amount of prodrug encapsulated, the optical fluorescamine assay was used (Figure S3a).^31^ The analysis was performed first by detecting the nonencapsulated 5-ALA present in the dialysis water and then by calculating the encapsulated amount by difference. The values of EE (%) and LC (%) are reported in Table 2. Three feeding amounts (0.62, 1.25, and 2.5 mg of 5-ALA) were used and a similar trend was detected with both nanosystems: as the feeding amount increased the EE decreased as the system was likely close to the maximum hostable amount and the concentration gradient was not able to boost the encapsulated amount. Indeed, by doubling the feeding amount the EE decreased from about 75% to 55% and 47%. On the other hand, the LC progressively increased from about 5% to 9% and 11%, as it expresses the mass of the encapsulated drug over the total mass of the sample. Thus, analyzing the lipid-to-drug ratio (that is, 4, 8, and 16 at the feeding mass of the drug equal to 0.62, 1.25, and 2.5 mg, respectively) for both types of vesicles, an increase of the EE can be observed as this ratio increases. Indeed, the EE shifts from about 47 to 56 and 76% as the lipid-to-drug ratio increases from 4 to 8 and 16, respectively.

**Table 2:** EE (%) and LC (%) of 5-ALA of the Lecithin/DLPC/Limonene and Lecithin/cisPC/Limonene Nanosystems as a Function of the Amount of 5-ALA Added

	EE (%)			LC (%)		
5-ALA mass (mg)	0.62	1.25	2.5	0.62	1.25	2.5
lecithin/DLPC/limonene	72 ± 10	55 ± 8	47 ± 3	4.6 ± 0.8	8.4 ± 1.8	11.2 ± 3.2
lecithin/cisPC/limonene	80 ± 11	57 ± 4	47 ± 1	5.2 ± 1.1	9.5 ± 3.1	12.5 ± 2.9

The encapsulation of 5-ALA led to a slight increase in the hydrodynamic diameter of the nanovesicles that was around 150 nm, as reported in Figure S2a. The spherical morphology of the nanovesicles under an electron microscope is shown as well (Figure S2b). Upon preparation the aqueous suspension of the loaded nanovesicles was stored at 4 °C and used within one month upon preparation. Indeed, as shown in Figure S2a, the size of the invasomes was stable up to one month. The possible leakage of the encapsulated prophotosensitizer was estimated, and no free 5-ALA was detected.

### 3.3 Release Kinetic and Diffusion Profile of the Nanovesicles

In preliminary tests, the release studies were performed in conditions that simulate the acidic pH of the endolysosomal compartment and thus by incubating the nanovesicles loaded with 5-ALA in acidic phosphate buffer (pH 4.5) at 37 °C up to 4 h; the same test was performed at physiological pH. Then the samples were centrifuged through Amicon filters, and the fluorescamine assay was performed to estimate the free 5-ALA passed through the membrane. A negligible release occurred (Figure S3b). Indeed, after 4 h of incubation, less than 5% of 5-ALA was detected at both pH. These data suggested that the vesicles were stable and preserved their integrity upon acidification of the surrounding medium. Other external stimuli, such as enzymatic degradation, were used to trigger the release. Since enzymes and other biomolecules might interact with fluorescamine leading to false data, the release assays were then performed with fluorescently labeled invasomes.

Rhodamine 101 was loaded into the nanovesicles as fluorescent tracer, and due to its structure, it was expected that it was accommodated into the lipid bilayer. Upon encapsulation of the fluorophore the vesicles exhibited a lower average hydrodynamic size but a greater PDI (Figure S4a,c); the estimated %EE show that the vesicles containing cisPC had a slightly higher EE (78% vs 71% EE), likely due to the longer alkyl chain as compared to DLPC.

**Figure 2:**
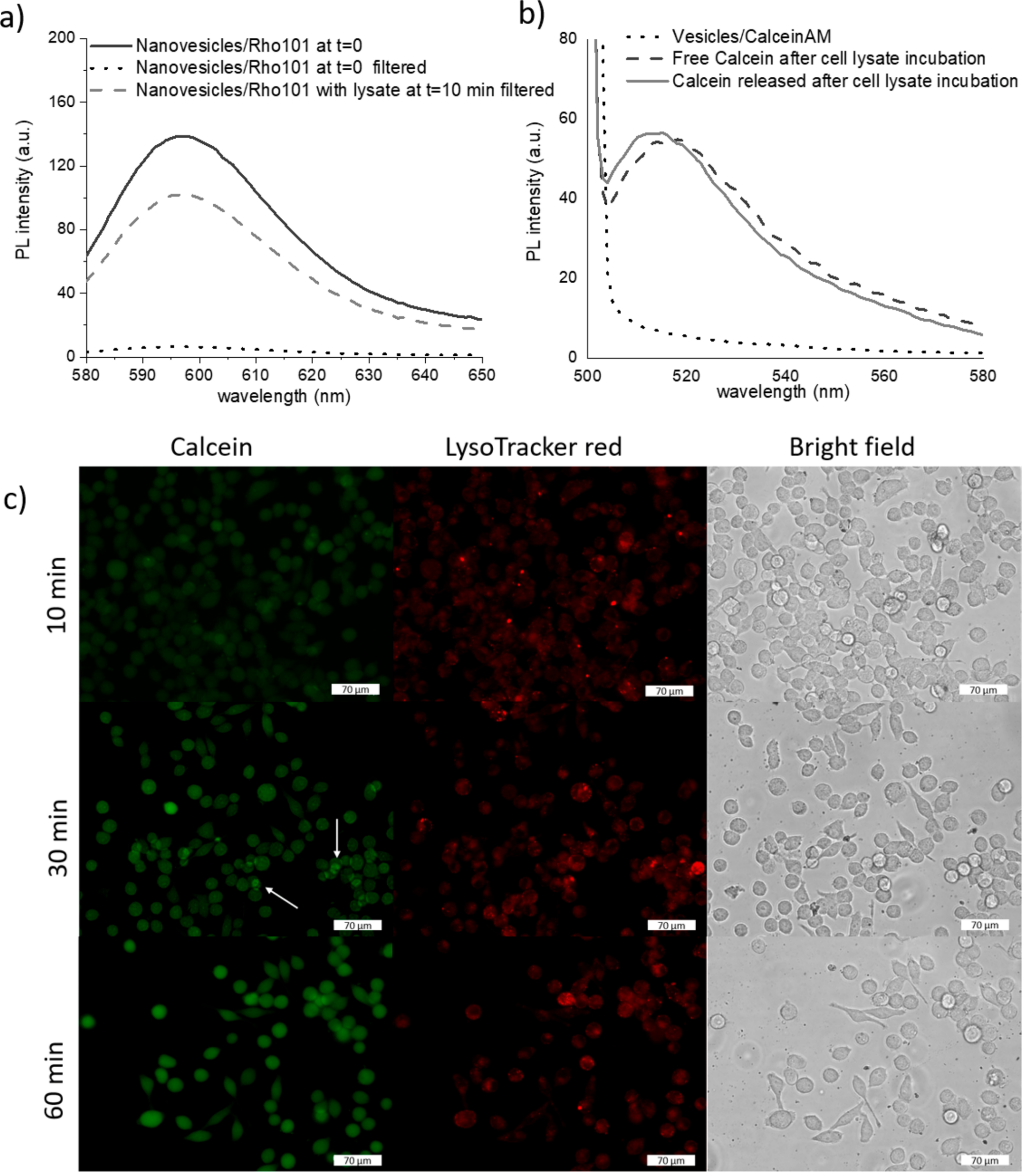
(a) Fluorescence spectra of the nanovesicles encapsulating rhodamine 101 before (black dotted curve) and after (gray dashed curve) enzymatic degradation with the cell lysates and filtration. The dark gray solid curve refers to the nanovesicles/Rho101 not filtered. (b) Fluorescence spectra of calcein upon enzymatic degradation with the cell lysates: the dotted curve refers to the nanovesicles loaded with calceinAM, while the solid curve is related to the calcein released from the nanovesicles upon enzymatic degradation. The dashed curve refers to free calcein upon lysate treatment. (c) Fluorescence microscopy images of melanoma cells incubated with the nanovesicles encapsulating calceinAM for 10, 30, and 60 min. The green fluorescent channel is associated with the released calcein and the red fluorescent channel to Lysotracker red staining. Scale bar is 70 μm.

Then, an enzymatic degradation test was performed. The fluorescent vesicles were indeed incubated with cellular lysates of melanoma cells, and after 10 min of incubation at 37 °C, the suspensions were filtered and the collected solutions were measured at the fluorimeter. The spectra reported in Figure 2a show the curves of the fluorescent nanovesicles without any enzymatic treatment prior (gray solid line) and after (gray dotted line) filtration with a 0.2 μm syringe filter: most of the fluorescence was retained by the filter (around 95%). This demonstrates that the intact nanovesicles did not pass through the filter. On the other hand, upon incubation for 10 min with the cell lysates and successive filtration, a fluorescence signal (light gray dashed line) was detected and was associated with the fluorophore released by the digested nanovesicles. By plotting the fluorescence signal on a calibration curve of rhodamine 101, a 70% release was estimated. When the incubation time was prolonged to 20 and 30 min, any additional release was detected.

To confirm these findings and that the fluorescence detected was derived from the degraded vesicles, an additional test was performed with the nanovesicles encapsulated with calceinAM (Figure S4b,c). It is a fluorescent tracer whose fluorescence is activated by the enzymatic degradation of the cellular esterases. Thus, the vesicles loaded with calceinAM did not exhibit any fluorescence (gray dotted curve of Figure 2b). On the other hand, upon incubation of the samples with the cell lysates for 10 min at 37 °C, a fluorescence spectrum in the green region was detected (Figure 2b, gray solid curve). Interestingly, the fluorescent curve (dark gray dashed curve) derived from the treatment of free calceinAM with the cell lysate is slightly shifted compared to that of released calcein. It is likely that the released molecule is partially associated with other components of the vesicle, thus displaying a blue shift of about 5 nm.

The optical images (Figure 2c) of melanoma cells incubated with the vesicles/calceinAM show that after 10 min the fluorescent signal is negligible, while at 30 min the green signal of calcein is already visible inside the cells both as accumulated in the endolysosomes and released in the cell cytoplasm. The localization in the digestive organelles is confirmed by the red fluorescent tracking of the lysosomes. After 60 min, the fluorescence is almost completely diffused in the cells.

**Figure 3:**
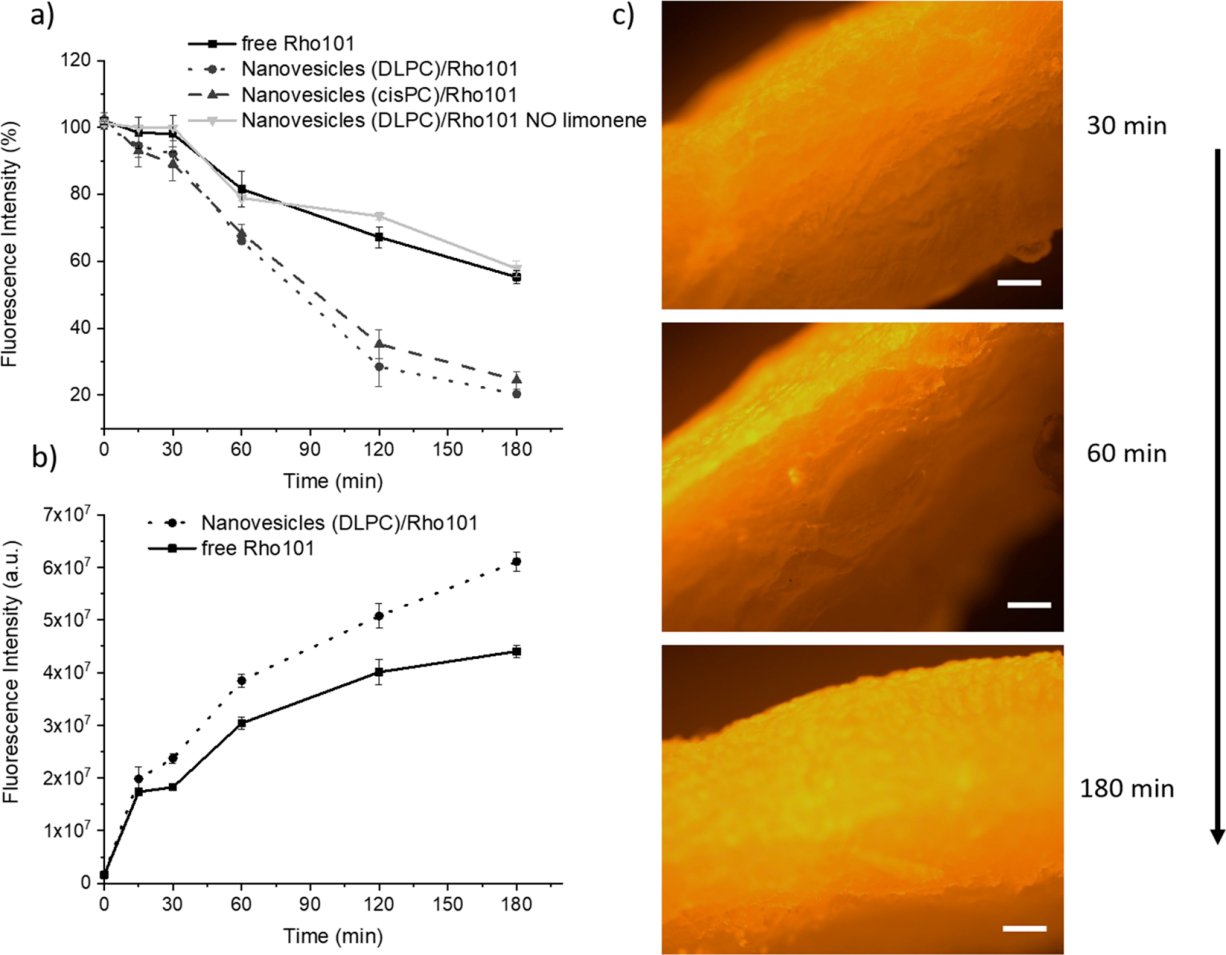
(a) Fluorescence intensity (%) of the solution containing either the fluorescent nanovesicles or the free dye measured upon incubation with the ear pig skin up to 3 h at 37 °C. (b) Fluorescence intensity (a.u.) released from the ear pig skin samples after the incubation with either the nanovesicles (lecithin/DLPC/limonene) or free rhodamine up to 3 h at 37 °C and upon dialysis for 24 h at 4 °C. (c) Optical images of the pig skin sections after incubation with the vesicles (lecithin/DLPC/limonene) for 30, 60, and 180 min. Scale bar = 200 μm.

Furthermore, to assess the potential use of the nanovesicles for PDT of skin cancer, a diffusion test with ear pig skin was performed; the decrease of the fluorescence signal of the nanovesicles/Rho101 was monitored over time up to 3 h at 37 °C. Free rhodamine 101 was also used as a control sample. As shown in Figure 3a, the nanovesicles exhibited a faster diffusion than free rhodamine: indeed, after 1 h, the fluorescence of the sample incubated with the nanovesicles decreased by 34%, while in the case of free rhodamine it decreased by 19%. At 3 h of incubation, about 20% of the fluorescence was detected in the case of the nanovesicles as compared to the free fluorophore (around 45%). This demonstrates the enhanced penetration of the nanovesicles through the skin compared to the free molecule. Furthermore, the diffusion of the fluorescent nanovesicles prepared without limonene was evaluated as well. Interestingly, the diffusion was much slower as compared to those including the terpene: after 3 h, indeed, the fluorescence signal detected was higher than 55%. These data confirm the enhanced penetration of the invasomes thanks to the presence of limonene that acts as a dermal penetrator.^32^ Furthermore, soon after the incubation time, the pig ears were collected, cut, and dialyzed against water, and the fluorescent dye (both free and encapsulated rhodamine) released from the samples was measured, as reported in Figure 3b. The trend of the two curves shows an incremental release over time and a higher fluorescence signal detected in the case of the invasomes.

The ear skin samples were also processed and analyzed under a fluorescence microscope (Figure 3c). The images of the skin slices show the diffusion of the fluorescent signal of rhodamine through the epidermidis over time. It can be observed that the penetration depth of the nanovesicles increased from 100 μm at 30 min up to 500 μm after 3 h incubation. The release and diffusion studies were performed with both types of nanovesicles (either with DLPC or cisPC) and no difference was detected. The images of Figure 3c refer to the sample containing cisPC. Based on the evidence that the presence of either DLPC or cisPC did not affect the average size of the resulting vesicles as well as the EE efficiency of the prodrug, the diffusion kinetics, and the degradation trend, the subsequent cellular studies to evaluate the therapeutic potential of nanovesicles loaded with 5-ALA were performed only with the vesicles containing DLPC.

### 3.4 Therapeutic Potential of the Nanovesicles Loaded with 5-ALA

**Figure 4:**
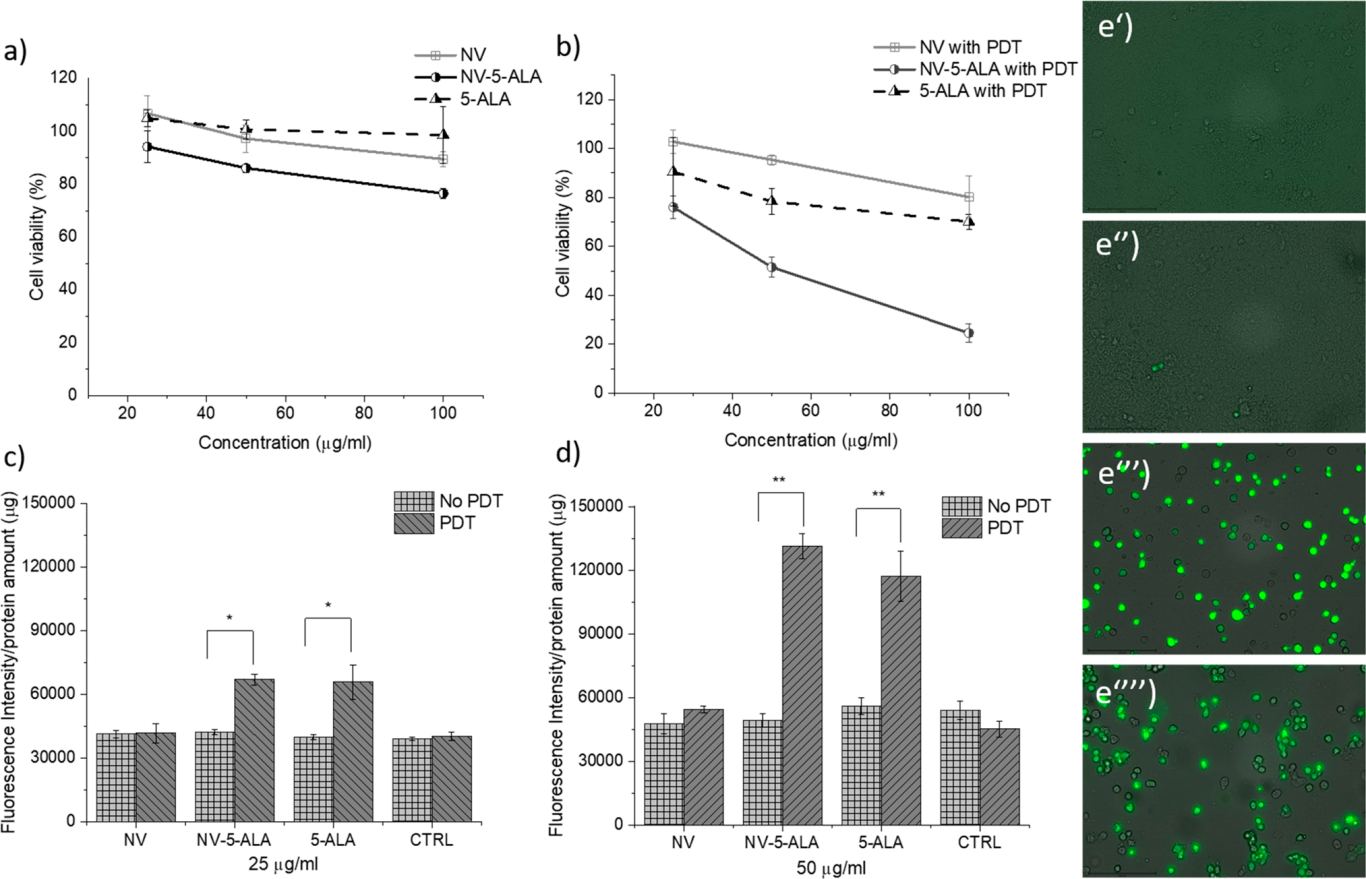
(a) MTT assay of HBL cells administered with free 5-ALA, empty NV, and NV-5-ALA for 3 h and assayed after 24 h, respectively. (b) MTT assay of HBL cells that received the nanovesicles and the photodynamic treatment (PDT). (c, d) DCF assay of HBL cells administered with free 5-ALA, empty NV, and NV-5-ALA for 3 h without and with PDT, respectively. Statistical analysis via *t* test (*p < 0.05; **p < 0.01). (e′–e′′′′) Representative fluorescent images of HBL cells after DCF assay: (e′) control cells; (e′′) cells incubated with plain vesicles; (e′′′ and e′′′′) cells administered with NV-5-ALA and free 5-ALA for 3 h and then irradiated, respectively.

To study the therapeutic potential of the nanovesicles loaded with 5-ALA and the response to the photodynamic treatment, we performed cellular tests with HBL melanoma cells. Both 2D and 3D cultures were used. In the experiments with 2D cultures, the melanoma cells were incubated with the 5-ALA-loaded nanovesicles at different concentrations (from 25 to 50 and 100 μg/mL); after 3 h of incubation, they were irradiated with the lamp (excitation wavelength at 630 nm) for 15 min before being assayed. Then MTT, Live/Dead, and DCF assays were performed to evaluate the impact of 5-ALA delivery combined with the PDT treatment on the cell viability and the onset of oxidative stress inside the cells. The MTT assay would provide information about the average metabolic response of the cells to the treatment, while the DCF assay would clearly indicate a condition of cellular stress related to the generation of ROS. Indeed, according to the reported mechanism of action, 5-ALA, once internalized and released in the cytosol, should be metabolized to protoporphyrin IX that upon light activation should induce the generation of oxygen radicals with consequent stress and cell death. Figure 4 shows the results obtained with the two assays. In the case of the MTT assay, Figure 4a and b report the viability trend of the cells treated with either the nanovesicles or the free molecule with and without the application of lamp irradiation, respectively. The irradiation induced a dramatic decrease in cell viability. In the case of the cells administered with free 5-ALA at the highest concentration (100 μg/mL) the viability decreased from 98% to 75%. The cytotoxic effect was much more intense when 5-ALA was loaded into the nanovesicles, as the viability dropped from around 80% to 25% at the highest concentration administered. At 50 μg/mL the viability reached about 50% upon photoirradiation. On the other hand, in the case of the plain vesicles, the viability was not affected by the light treatment. Therefore, it looks that 5-ALA delivered by the nanovesicles is more efficient to induce cell death, likely due to an enhanced intracellular transport.

In the case of the DCF assay (Figure 4c,d), the cells were incubated with 5-ALA at 25 and 50 μg/mL, either free or encapsulated. The combined treatment of the cells with 5-ALA, either free or encapsulated, and PDT provoked strong ROS generation in HBL cells. This effect is 2 times higher than in the cells administered with plain nanovesicles. In addition, the generation of ROS species almost doubled by doubling the administered amount of 5-ALA. Representative fluorescent images of the cells that underwent PDT after the DCF assay are reported in panels e′–e′′′′ of Figure 4. While control cells and those incubated with the plain vesicles show healthy cells without fluorescent spots, the samples incubated with 5-ALA, either free or encapsulated, display green and rounded cells, a clear sign of cellular sufferance, detachment from the substrate, and a propensity to die. The two assays were also performed with the human keratinocyte cell line, Hacat. Cells were incubated with the nanovesicles and assayed accordingly. The data are reported in Figure S5 and evidence that the keratinocytes are not sensitive to the treatment with 5-ALA coupled to the light irradiation. Indeed, in the case of the MTT assay, the viability did not decrease below 80% as compared to control cells in any of the conditions tested (Figure S5a,b). Similarly, the DCF assay shows that the administration of 5-ALA followed by PDT did not induce the generation of ROS species. The fluorescence signal detected is very weak as confirmed by the fluorescent images of the cells (panels e′–e′′′′).

**Figure 5:**
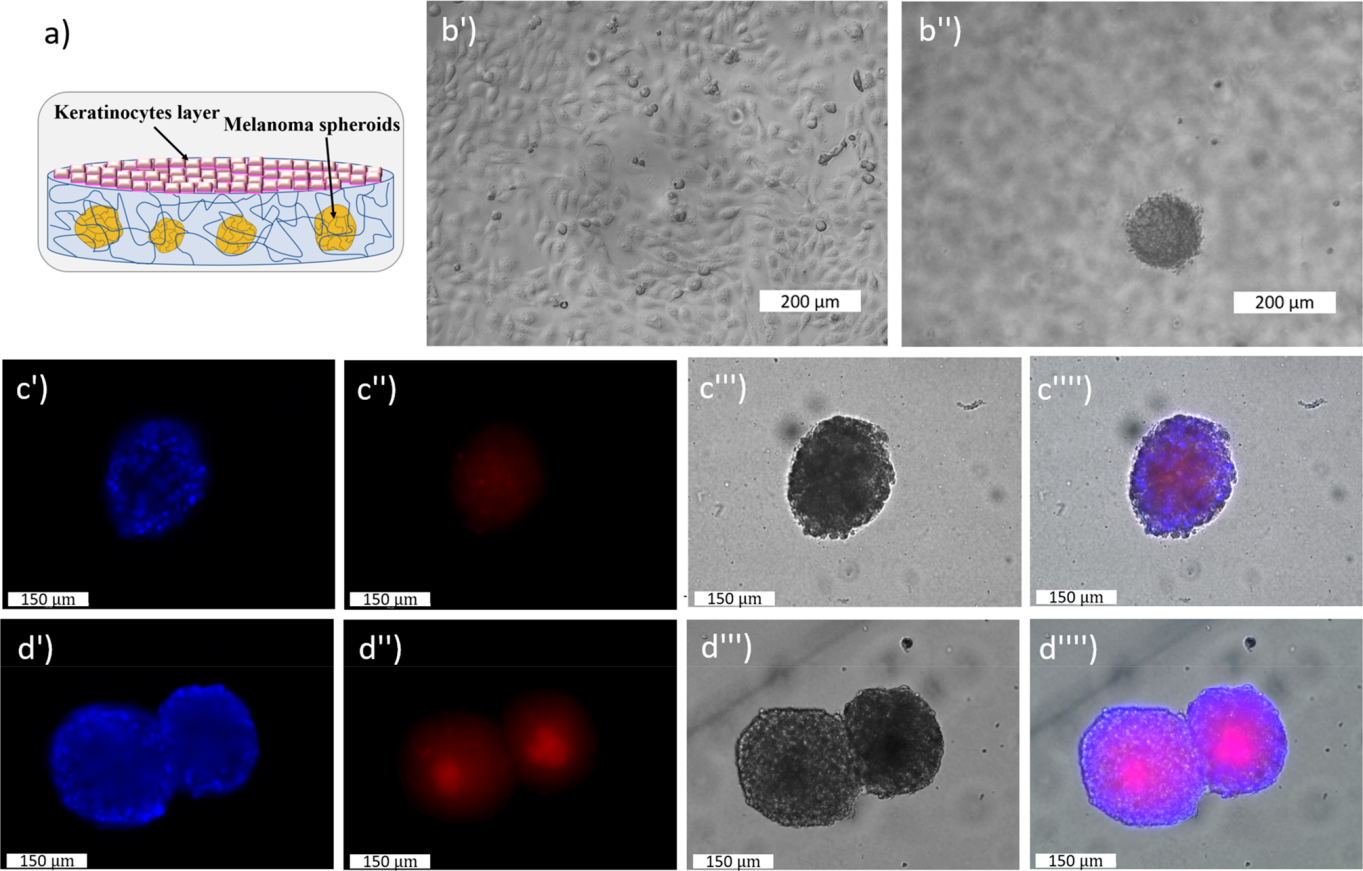
(a) Sketch of the hydrogel scaffold used for the growth of the melanoma spheroids and the formation of the keratinocytes monolayer. (b′, b′′) Optical images of the Hacat cell monolayer on top of the hydrogel and the embedded melanoma spheroids in the same *x*–*y* position but at a different *z*-plane, respectively. (c′–c′′′′ and d′–d′′′′) Fluorescent images of the melanoma spheroids embedded into the agarose hydrogel and incubated with NV-Rho101 for 1.5 and 3 h, respectively. The nuclei were stained with DAPI.

Furthermore, the biological assays were also performed with melanoma three-dimensional (3D) spheroids grown into a hydrogel matrix coated by a confluence monolayer of keratinocytes.^33^ The use of a simplified 3D scaffold-based skin model has been chosen to evaluate the diffusion and the therapeutic effect of the nanovesicles in a condition that mimics, more than a simple 2D culture, a tissue-like environment, although we are aware that the hydrophobicity of the scaffold employed for melanoma spheroids growth is not exactly the same as that displayed by human skin. The sketch of Figure 5 depicts the architecture of the 3D scaffold containing the melanoma spheroids coated by Hacat keratinocytes, while panels b′ and b′′ show the distribution on different *z*-planes of the keratinocytes (upper focal plane) and of the tumor spheroids (lower focal plane). First, the diffusion of the invasomes into the hydrogel was evaluated. Once the spheroids reached an average diameter of 150 μm, and after the formation of the keratinocyte monolayer the nanovesicles-Rho101 were added and left under incubation for 1.5 and 3 h. Then, the samples were fixed and the nuclei stained with DAPI. The images of Figure 5c (1.5 h) and d (3 h) evidence the accumulation of the fluorescent signal inside the spheroids. These findings confirm the time dependent uptake of the invasomes inside the embedded 3D cultures.

**Figure 6:**
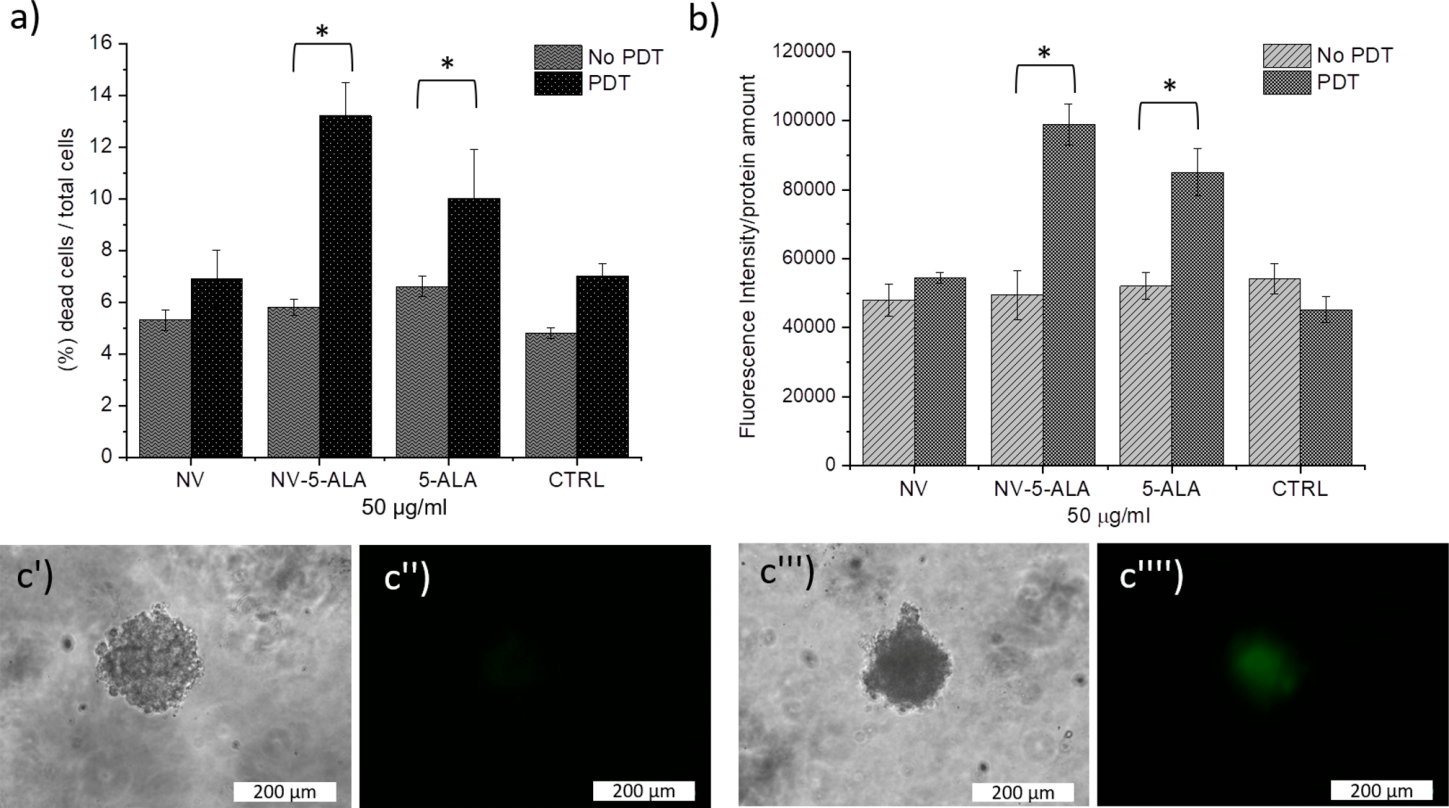
(a) Percentage ratio of dead cells/total cells estimated by the Live/Dead assay normalized versus the protein content. The assay was performed with the hydrogel-embedded melanoma spheroids incubated with either free or encapsulated 5-ALA without and with exposure to phototreatment. (b) DCF assay of HBL spheroids embedded into agarose hydrogels administered with free 5-ALA, empty NV and NV-5-ALA for 3 h without and with PDT, respectively (*p < 0.05). (c) Representative bright field and fluorescent images of melanoma spheroids (c′ and c′′, control samples, c′′′ and c′′′′, spheroids incubated with the NV-5-ALA and light-irradiated) after a DCF assay, respectively.

Then, Live/Dead and DCF assays were performed with the melanoma spheroids incubated with the nanovesicles and 5-ALA after exposure to the phototreatment. By the Live/Dead assay, the percentage ratio of dead cells over the total number of cells was estimated within each sample. Without the photoirradiation, the percentage ratio is similar in all the spheroids, regardless of the treatment they underwent (Figure 6a). On the other hand, after exposure to the light, the percentage ratio increased in the samples incubated with 5-ALA and NV-5-ALA. The latter shows a significant 2× increase of the dead cells as compared to the nonirradiated samples. Furthermore, the DCF assay was performed upon incubation of the agarose-embedded spheroids with the invasomes. The results of this assay (Figure 6b) clearly show that there is an effect of the combined 5-ALA/light treatment on the generation of intracellular ROS. It looks that both the free and the encapsulated prophotosensitizer induce a significant increase of the ROS species upon application of the light. Indeed, the normalized fluorescence signal is doubled in the case of the samples incubated with the loaded nanovesicles and free molecules upon light irradiation. On the other hand, without any phototreatment, the measured ROS values are almost similar for all the samples. Noteworthy, the effect obtained with the loaded nanovesicles is only slightly higher (but not significantly different) than that of free 5-ALA: this finding might diminish the potential benefits derived by the use of the nanovector. Nevertheless, it is important to underline that the hydrogel matrix employed for these preliminary studies does not represent a realistic model of the skin structure and thus does not enable testing the delivery capability in a hydrophobic environment. These preliminary studies were devised to assess the feasibility of the delivery approach in 3D melanoma cultures.

## 4 Discussion

Topical treatment of benign forms of tumors is an efficient and alternative approach to limit diffuse side effects of systemic drug administration and to reduce the drug dosage. The photodynamic therapy represents a well-established modality for the treatment of skin lesions and has been successfully used to kill cancer cells, such as basal cell carcinoma and nonmetastatic melanoma.^5,34,35^

PDT involves the use of a photosensitizer that under light irradiation reaches the excited state and transfers energy to the surrounding oxygen-generating radicals that in turn oxidize nucleic acids, proteins, and phospholipids.^8,36^ This cascade finally leads to cellular damage up to death. 5-ALA is the precursor of the synthesis of the endogenous photosensitizer Protoporphyrin IX; it is currently used in dermatology and for the treatment of accessible skin cancers.^12,16,37^ One of the main limitations of 5-ALA, a hydrophilic zwitterion, is the poor penetration through the hydrophobic layers of skin.^38^ To face this limitation, the use of a lipid-based nanocarrier may represent a good solution to accelerate the diffusion as well as the penetration of the prodrug through the epidermis and enhance the intracellular localization. So far, several types of lipid-based nanosystems have been developed, from classic to ultradeformable liposomes,^22,39^ from coloaded liposomes to ethosomes.^23,27^

In this study, egg lecithin-based invasomes containing the terpene limonene were prepared and optimized to encapsulate 5-ALA. Indeed, preliminary tests were performed to define the composition and preparation parameters: the combination that provided the highest biocompatibility and the smallest size was chosen. Two other phospholipids, either DLPC or cisPC, were used as additional components to obtain nanovesicles with an average size smaller than 150 nm, a diameter considered suitable for overcoming the skin barrier.^40^ In addition, the presence of the terpene provides higher elasticity to the whole system.^24^ The nanovesicles looked very stable: the average diameter, PDI, and thermal stability were monitored up to 30 days and did not evidence any significant variation. A pH gradient was exploited to improve the encapsulation efficiency of the prodrug in the aqueous core: this allowed us to increase the amount of 5-ALA encapsulated by almost 5 times.

The degradation profile of the nanovesicles upon enzymatic activity and diffusion was assessed by tracking the fluorescence of rhodamine and calcein. A rapid degradation (within 30 min in cellular studies), a 70% diffusion, and about 500 μm in depth penetration after 3 h incubation through the pig skin were detected. On the other hand, free rhodamine was less effective to cross the skin (close to 50%). Notably rhodamine displays a logP close to 1.8, while 5-ALA is equal to −1.5. Thus, Rhodamine is much more hydrophobic than 5-ALA that reasonably should show a much lower diffusion profile through the skin in comparison to the free fluorophore. In addition, as reported in the skin diffusion test, the presence of the terpene enhanced the penetration of the invasomes: indeed, the vesicles prepared without limonene show a slower diffusion (similar to that of free Rhodamine) as compared with the invasomes. These findings confirmed the good performance of the nanovesicles as skin diffusion enhancers of aqueous molecules and that the presence of the terpene boosts their skin permeability. This feature is particularly relevant for 5-ALA use as an effective prophotosensitizer.

Finally, *in vitro* studies were performed on cellular human models of melanoma. Classical 2D melanoma cell cultures, treated with 5-Ala free or encapsulated into nanovesicles, allowed to verify not only the biocompatibility of nanovesicles, but also the timing and efficiency of PDT in causing the alteration of cellular homeostasis. The viability and the ROS detection assays showed that the treatment with the encapsulated prophotosensitizer was significantly more efficient than the free 5-ALA in terms of both penetration and delivery to trigger ROS generation and induce cell death. On the other hand, the same tests performed with Hacat cells, a human keratinocytes line, showed a reduced effect of the combined 5-ALA/PDT treatment on nontumor cells, thus supporting the good biocompatibility of the vesicles versus healthy cells. These findings are in accordance with previous studies that reported about the enhanced sensitivity of cancer cells to 5-ALA treatment due to the accelerated metabolization of the prophotosensitizer to the protoporphyrin IX.^41^

Furthermore, the diffusion and the therapeutic effect of the nanovesicles have been also studied in a simplified 3D scaffold-based skin model in which melanoma spheroids were grown embedded into an agarose scaffold coated by layered keratinocytes. The 3D culture tests showed that the invasomes were able to penetrate the keratinocyte layer as well as the hydrogel matrix, thus easily reaching the core of spheroids, deliver 5-ALA and, after phototreatment, cause cell death. Starting from our outcomes, future *in vivo* studies will be addressed in order to evaluate pharmacokinetics and tissue distribution of 5-ALA invasomes and prove their therapeutic efficacy.

## Supporting Information Available

Average DLS size of the nanovesicles containing the terpenes (Table S1); Phospholipid molecules tested as components of the invasomes (Table S2); TGA curves of the nanovesicles after 30 days storage (Figure S1); Average DLS size of the 5-ALA nanovesicles at time 0 and after 30 days storage at 4 °C (Figure S2); Release assay of 5-ALA at pH 7.4 and 4.5 (Figure S3); DLS size curves of the two nanosystems loaded with rhodamine 101 and calceinAM (Figure S4); MTT and DCF assay of Hacat cells administered with the nanovesicles (Figure S5) (PDF)
