# Supplementary material for: Enhanced Delivery of 5-Aminolevulinic Acid by Lecithin Invasomes in 3D Melanoma Cancer Model
Source: Mol Pharm. 2023 Sep 27;20(11):5593–606. doi: 10.1021/acs.molpharmaceut.3c00494 (PMC10630953; doi:10.1021/acs.molpharmaceut.3c00494)
Supplement: Supplementary file 1 — mp3c00494_si_001.pdf [file mp3c00494_si_001.pdf]

SUPPORTING INFORMATION

Enhanced delivery of 5-aminolevulinic acid by lecithin invasomes in 3D melanoma cancer model

*Antonio Gaballo[#, *], Andrea Ragusa[&], Concetta Nobile[#], Nunzia Gallo[$], Luca Salvatore[§], Clara Piccirillo[#], Alessia Nito[#], Annalisa Caputo[#], Gabriella Guida[%], Alfredo Zito[∞], Raffaele Filotico[£,†] and Alessandra Quarta[#, *]*

[#] Consiglio Nazionale delle Ricerche, Institute of Nanotechnology, via Monteroni, 73100 Lecce, Italy.

[&] Department of Biological and Environmental Sciences and Technologies, University of Salento, via Monteroni, 73100 Lecce, Italy.

[$] Department of Engineering for Innovation, University of Salento, via Monteroni, 73100 Lecce, Italy.

[§] Typeone Biomaterials Srl, Muro Leccese, (Lecce), 73036, Italy.

[%] Department of Basic Medical Sciences Neurosciences and Sense Organs, University of Bari, Bari, 70124, Italy.

[∞] Pathology Department, IRCCS Istituto Tumori “Giovanni Paolo II”, Bari, 70124, Italy.

[£] Dermato-Oncology Unit, IRCCS Istituto Tumori “Giovanni Paolo II”, Bari, 70124, Italy.

[†] Section of Dermatology and Venereology, Department of Precision and Regenerative Medicine and Ionian Area (DiMePRe-J), University of Bari “Aldo Moro”, Bari, 70124, Italy.

Table S1. Average DLS size and PDI values of the egg lecithin-based nanovesicles containing the terpenes at three concentrations (1, 2.5 and 5 mg). The last column reports the IC_{50} values obtained by MTT assay of melanoma cells incubated with the lecithin-terpene nanovesicles for 24 h.

Mass (mg)	DLS size (nm)			PDI			IC_{50} on HBL cells		
	Eugenol	Geraniol	Limonene	Eugenol	Geraniol	Limonene	Eugenol	Geraniol	Limonene
**1**	320 ± 5	282 ± 1	256 ± 1	0.15	0.21	0.17	640 ± 38	520 ± 43	595 ± 9
**2.5**	294 ± 3	254 ± 3	241 ± 4	0.22	0.23	0.17	241 ± 25	329 ± 28	303 ± 12
**5**	237 ± 3	205 ± 2	218 ± 3	0.24	0.21	0.13	98 ± 11	140 ± 27	165 ± 18

Table S2. Phospholipid molecules tested and added to the other components of the invasomes (lecithin and limonene). The average size and the IC_{50} estimated by MTT assay of melanoma cells are reported in the third and fourth column, respectively.

**Lipid molecules tested**	**Length of the alkyl chain/unsaturation**	**Average size of the vesicles (nm)**	**IC_{50} on HBL cells**
**1,2-dilauroyl-sn-glycero-3-phosphocholine (DLPC)**	12:0	125 ± 7	495 ± 21
**1,2-dimyristoyl-sn-glycero-3-phosphocholine (DMPC)**	14:0	180 ± 11	530 ± 16
**14:0 1-myristoyl-2-hydroxy-sn-glycero-3-phosphocholine (Lyso PC)**	14:0	230 ± 6	238 ± 12
**1,2-Distearoyl-sn-glycero-3-phosphorylethanolamine (DSPE)**	18:0	250 ± 14	283 ± 14
**1,2-dioleoyl-sn-glycero-3-phosphocholine (CisPC)**	18:1	145 ± 1	530 ± 34

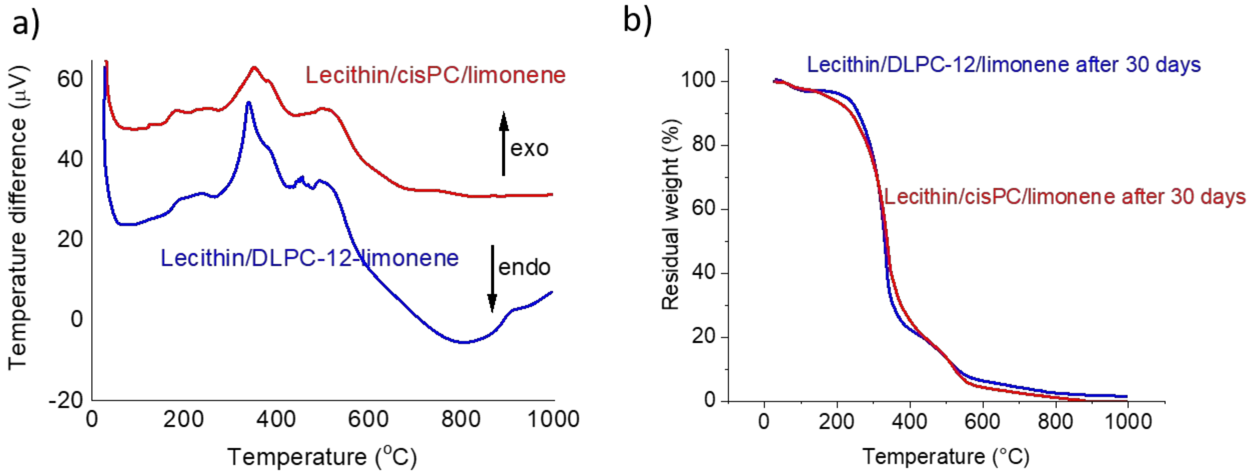

Figure S1. a) DSC curves of the two nanosystems; b) TGA curves (Percentage residual weight) of the nanovesicles containing DLPC-12 and cisPC after 30 days storage at 4°C.

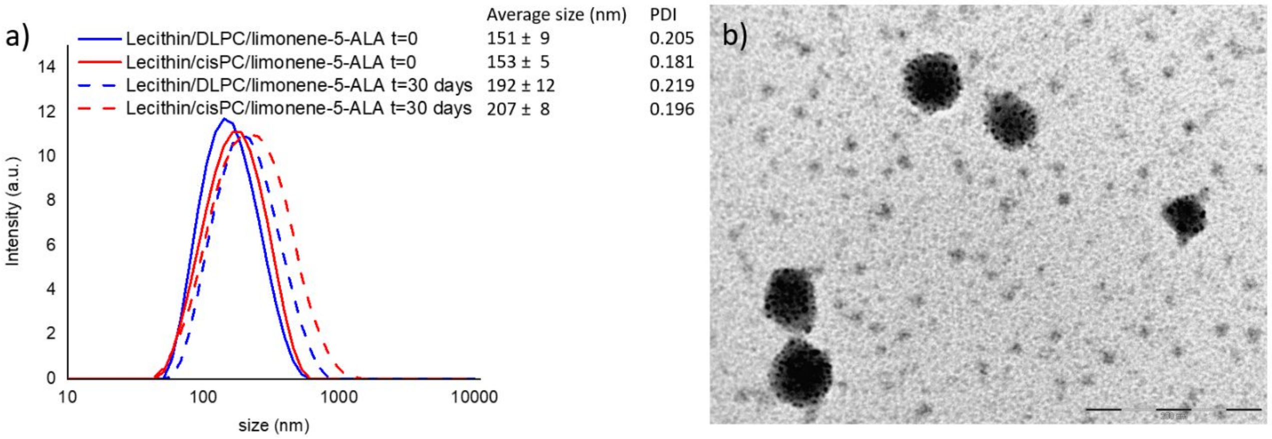

Figure S2. a) DLS curves, average hydrodynamic size and PDI of the two types of nanovesicles loaded with 5-ALA at time 0 and after 30 days storage at 4°C. b) TEM image of the nanovesicles prepared with DLPC and loaded with 5-ALA (scale bar corresponds to 200 nm).

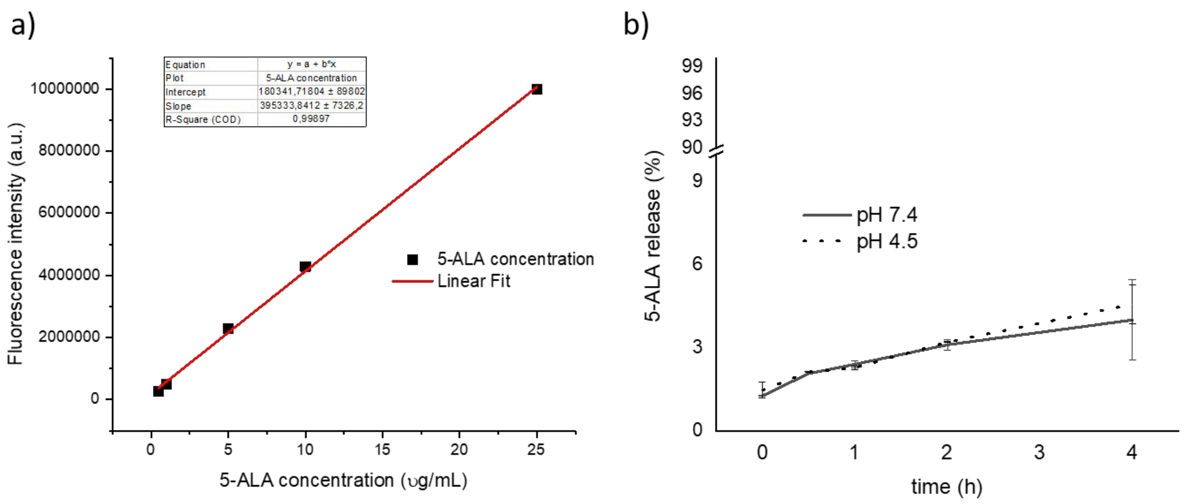

Figure S3. a) Calibration curve of 5-ALA based on Fluorescamine assay. b) Release assay of 5-ALA from the nanovesicles at pH 7.4 and 4.5 up to 4 h incubation at 37 °C.

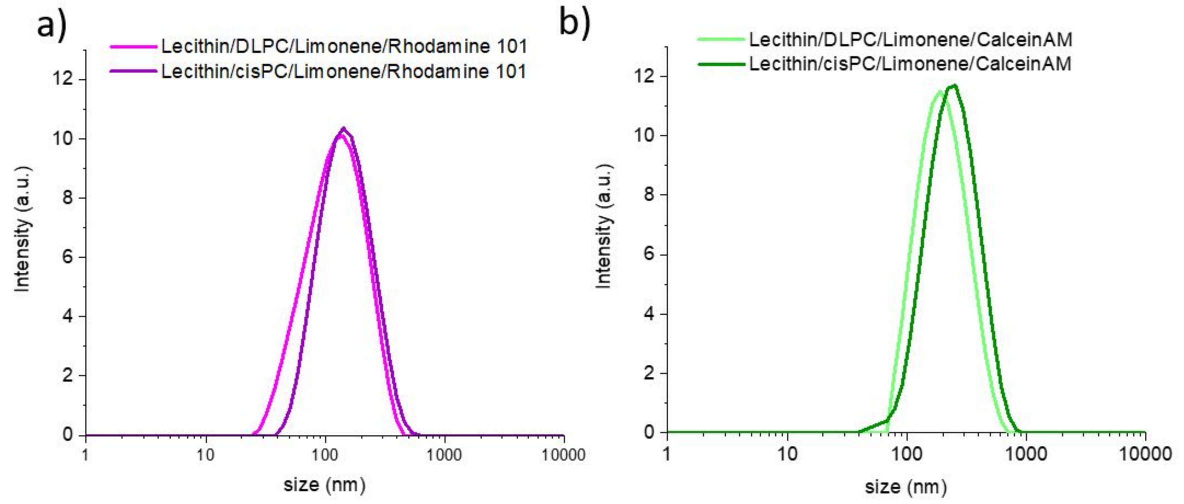

c)

Sample	DLS size (nm)	PDI	%EE
Lecithin/DLPC/limonene/Rhodamine 101	130 ± 1	0.219	71 ± 8
Lecithin/cisPC/limonene/Rhodamine 101	116 ± 1	0.229	78 ± 5
Lecithin/DLPC/limonene/CalceinAM	167 ± 2	0.213	/
Lecithin/cisPC/limonene/CalceinAM	210 ± 5	0.185	/

Figure S4. a-b) Size curves of the two nanosystems loaded with Rhodamine 101 and CalceinAM, respectively, as measured by DLS. c) Average hydrodynamic diameter, PDI and percentage encapsulation efficiency of the two types of nanovesicles loaded with the two fluorophores.

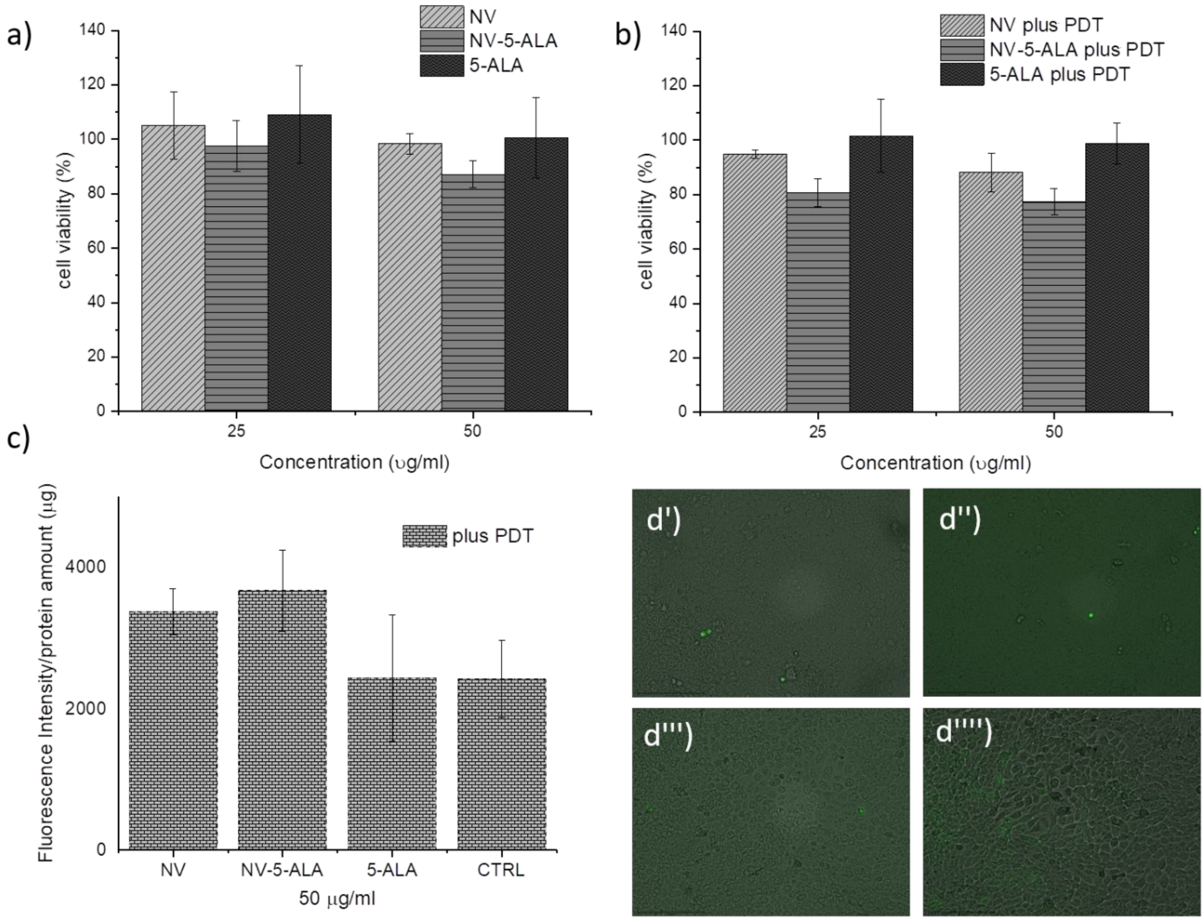

Figure S5. a) MTT assay of Hacat cells administered with free 5-ALA, Empty NV and NV-5-ALA for 3 h and assayed after 24 h, respectively. b) MTT assay of Hacat cells that received the nanovesicles and the photodynamic treatment (PDT). c) DCF assay of Hacat cells administered with free 5-ALA, Empty NV and NV-5-ALA for 3 h plus PDT. d'-d'''') Representative fluorescent images of Hacat cells after DCF assay. d') cells incubated with plain vesicles; d'') cells administered with NV-5-ALA; d''') cells administered with free 5-ALA; d'''') control cells, respectively.
